# Comparing Professional and Consumer Ratings of Mental Health Apps: Mixed Methods Study

**DOI:** 10.2196/39813

**Published:** 2022-09-23

**Authors:** Georgie Hudson, Esther Negbenose, Martha Neary, Sonja M Jansli, Stephen M Schueller, Til Wykes, Sagar Jilka

**Affiliations:** 1 Institute of Psychiatry Psychology and Neuroscience King's College London London United Kingdom; 2 South London and Maudsley NHS Foundation Trust London United Kingdom; 3 Division of Psychology and Language Sciences University College London London United Kingdom; 4 Department of Psychological Science University of California Irvine, CA United States; 5 Department of Informatics University of California Irvine, CA United States; 6 Warwick Medical School University of Warwick Coventry United Kingdom

**Keywords:** well-being, apps, patient and public involvement, coproduction, mental health, service user, technology, mobile health, mHealth, digital, mobile phone

## Abstract

**Background:**

As the number of mental health apps has grown, increasing efforts have been focused on establishing quality tailored reviews. These reviews prioritize clinician and academic views rather than the views of those who use them, particularly those with lived experiences of mental health problems. Given that the COVID-19 pandemic has increased reliance on web-based and mobile mental health support, understanding the views of those with mental health conditions is of increasing importance.

**Objective:**

This study aimed to understand the opinions of people with mental health problems on mental health apps and how they differ from established ratings by professionals.

**Methods:**

A mixed methods study was conducted using a web-based survey administered between December 2020 and April 2021, assessing 11 mental health apps. We recruited individuals who had experienced mental health problems to download and use 3 apps for 3 days and complete a survey. The survey consisted of the One Mind PsyberGuide Consumer Review Questionnaire and 2 items from the Mobile App Rating Scale (star and recommendation ratings from 1 to 5). The consumer review questionnaire contained a series of open-ended questions, which were thematically analyzed and using a predefined protocol, converted into binary (positive or negative) ratings, and compared with app ratings by professionals and star ratings from app stores.

**Results:**

We found low agreement between the participants’ and professionals’ ratings. More than half of the app ratings showed disagreement between participants and professionals (198/372, 53.2%). Compared with participants, professionals gave the apps higher star ratings (3.58 vs 4.56) and were more likely to recommend the apps to others (3.44 vs 4.39). Participants’ star ratings were weakly positively correlated with app store ratings (*r*=0.32, *P*=.01). Thematic analysis found 11 themes, including issues of user experience, ease of use and interactivity, privacy concerns, customization, and integration with daily life. Participants particularly valued certain aspects of mental health apps, which appear to be overlooked by professional reviewers. These included functions such as the ability to track and measure mental health and providing general mental health education. The cost of apps was among the most important factors for participants. Although this is already considered by professionals, this information is not always easily accessible.

**Conclusions:**

As reviews on app stores and by professionals differ from those by people with lived experiences of mental health problems, these alone are not sufficient to provide people with mental health problems with the information they desire when choosing a mental health app. App rating measures must include the perspectives of mental health service users to ensure ratings represent their priorities. Additional work should be done to incorporate the features most important to mental health service users into mental health apps.

## Introduction

### Interest in Mental Health Apps

Digital technologies can expand access to mental health care. The availability of smartphone apps to support mental health and well-being has increased over the last few years, with some evidence supporting their use in depression [1], anxiety [2], and other mental health conditions [3]. A survey of interest in smartphone apps among military veterans found that 43% indicated an interest in using a mental health app [4]; however, despite this enthusiasm, only 11% had done so, with a major barrier to adoption including concerns around a lack of proof of efficacy (72%). This lack of proof is reflected in the rapid expansion of available mental health apps with little regulation or oversight [5-9]. This problem of “high availability but low evidence base” [10] means that many publicly available products have little or no evidence supporting their use.

### Consumer Preferences in Mental Health Apps

Consumers want access to clear information when choosing an app [11,12], and several measures have been developed to provide this information on the app of choice. The most commonly used measure is the Mobile App Rating Scale (MARS) [13], which assesses *engagement*, *aesthetics*, and *usability*. However, on its own, this measure does not provide enough information to allow service users to decide whether to use an app [14]. ORCHA, a for-profit company [15], reviews apps on *current standards*, *regulations*, and *good practices* but only provides a composite score, which does not allow service users to identify factor ratings that are most personally important to their choice. When consumers are choosing apps, often the only measures available are star ratings and reviews. These reviews may be written by genuine app users, but there are large numbers of fake reviews, which are hard to distinguish from genuine ones [16]. In the United Kingdom, the general population had increased experiences of insomnia, anxiety, low mood, and general psychological distress during the COVID-19 pandemic and subsequent lockdowns [17-19]. There was also a 200% increase in mental health app use [20], with many people relying on mental health care apps on their phones when their usual care was disrupted. Owing to this increased use, we need to understand what matters to consumers when selecting mental health apps to convey that information so they can make informed choices. A study found that people with mental health needs value aesthetics and data security when choosing a mental health app [21]; however, it is not known whether these values differ from professionals’ views.

### This Study

This study aimed to fill this gap by understanding how those with lived experience perceive mental health apps and how these views differ from clinicians’ and academics’ reviews and those provided on app stores. The findings will contribute to a broader understanding of consumers’ use of and opinions on mental health apps.

## Methods

### Design

This was a cross-sectional mixed methods study evaluating 11 mental health apps.

### Ethics Approval

The study received ethical approval from the King’s College London Psychiatry, Nursing and Midwifery Research Ethics Subcommittees on November 30, 2020 (LRS-20/21-21137). All participants provided written consent before participating.

### Patient and Public Involvement

We consulted the Young Person’s Mental Health Advisory Group [22] on the design of the study. This included the length of time participants should use each app and phrasing of questionnaire items to improve clarity.

### Participants and Recruitment

Participants were recruited using volunteer sampling from advisory groups, local mental health groups, and a general university-wide newsletter. Participants were included if they were aged ≥18 years, living in the United Kingdom, had access to a smartphone, were able to download smartphone apps, and had a history of mental health problems. Participants were not screened for psychiatric diagnoses but were asked whether they had “experience of mental health difficulties.” This was judged to be most appropriate in this study, as smartphone use is lower in those with serious mental illnesses [23,24], and most mental health apps target improving general well-being rather than severe symptoms.

### Apps

#### App Selection

We initially selected 12 apps for the trial. All apps had to be freely available for download, as consumers strongly prefer free apps [21]. Overall, 50% (6/12) of our initial apps were selected from the highest-ranking mental health apps on Google Play and iOS app stores, and these were supplemented with 6 of the highest-rated apps based on the One Mind PsyberGuide Credibility Rating Scale [25]. We removed 1 app as it required users to sign up for a free trial that would automatically upgrade to an annual subscription, reducing the number of apps to 11.

#### Overview of Apps

The 11 apps assessed were Breethe, Calm, Headspace, Insight Timer Meditation, MindDoc, MindShift, Reflectly, Remente, Sanvello, Self-Help for Anxiety, and Woebot. These apps can be divided into three categories: meditation (Calm, Headspace, Breethe, and Insight Timer Meditation), journaling (Reflectly, Remente, and MindDoc), and cognitive behavioral therapy (Woebot, MindShift, Sanvello, and Self-Help for Anxiety). Despite these categories, apps have a considerable overlap of functionality, as demonstrated in the study by Lagan et al [26]. For example, although based on cognitive behavioral therapy, MindShift, Sanvello, and Self-Help for Anxiety also use meditation and relaxation techniques. Therefore it is not possible to differentiate reviews based on app category.

### Professional Reviewers

Professional reviews were completed by a team of 4 highly trained raters. Training takes between 3 and 4 hours and consists of a video tutorial and app ratings, followed by in-person reliability checks against an expert rater.

### Measures

Textbox 1 presents the participant and professionals’ measures and app store ratings.

**Participants’ and professionals’ measures and app store ratings.**

**Participants’ measures**

- *Demographic information and app use*: Age, ethnicity, gender, education, employment status, average daily digital device use, and most frequently used digital device.
- *One Mind PsyberGuide Consumer Review Questionnaire*: 12 open-ended questions were derived from the metrics used by One Mind PsyberGuide, including the One Mind PsyberGuide credibility scale [25], Mobile App Rating Scale (MARS) [13], and One Mind PsyberGuide transparency scale [27]. These 12 questions were mapped onto six app domains:
  1. Ease of use (“How easy or hard was this app to use?”)
  2. Difficulties of use (“Were there any parts of the app that were confusing or difficult to use?”)
  3. Engagement (“Did you enjoy using this app?”)
  4. Aesthetics (“What did you think about how this app looked?”)
  5. Perceived impact on well-being (“What impact, if any, did this app have on your well-being?”)
  6. Data security (“Did you feel confident that the data you entered in this app was secure?”)

- *MARS* [13]: We used two items from the MARS based on recommendations by our service user advisers:
  1. *Recommendation ratings*: If they would recommend this app to people who might benefit from it on a 5-point Likert scale.
  2. *MARS star ratings*: A star rating from 1 to 5.

**Professionals’ measures**

Professionals’ ratings of all apps were collected from the One Mind PsyberGuide website. The data were as follows:

- *MARS* [13]: Professionals’ recommendations and MARS star ratings from 1 to 5 were assessed. This measure also captured professionals’ ratings of the domains of app functionality, engagement, aesthetics, and perceived impact on well-being, which were mapped onto the participants’ ratings. These were measured using a 5-point Likert scale.
- *PsyberGuide Transparency Score*: Professionals’ ratings of the presence and quality of a privacy policy were used. This measure comprises 7 subquestions and results in a binary classification of data security (acceptable or unacceptable). This measure was used previously [27] and adapted from the Enlight evaluation tool [28].

**App store ratings**

- Average star ratings for each app were collected from both iOS and Google Play stores on November 19, 2021, and the scores were averaged across both app stores.

### Procedure

Once participants had consented, they were randomly allocated 3 of the 11 apps. They used the 3 apps over 3 days, with a total participation period of 3 days, as suggested by our service user advisers. This also corroborates previous work, which found that the number of times a mental health app is opened declines by 80% over the first 10 days of use [29]. Participants were encouraged to explore the features of the apps and to use the apps for 10 to 60 minutes per day, spending an equal amount of time on each app. On the evening of the third day, participants completed the MARS ratings and the One Mind PsyberGuide Consumer Review for each app via SurveyMonkey.

We compared reviews of consumers and professionals on the following six domains:

1. Ease of use (“How easy or hard was this app to use?”)
2. Difficulties of use (“Were there any parts of the app that were confusing or difficult to use?”)
3. Aesthetics (“What do you think about how this app looked?”)
4. Engagement (“Did you enjoy using the app? (eg, was it engaging, fun, or boring?)“)
5. Perceived impact on well-being (“What impact, if any, did this app have on your well-being?”)
6. Data security (“Did you feel confident that the data you entered in this app were secure? Why, or why not?”)

### Data Analysis

#### Quantitative

We converted the qualitative text from the One Mind PsyberGuide Consumer Review into a quantitative binary classification (“1” a positive experience and “0” a negative experience) using a predefined protocol (Multimedia Appendix 1). Two researchers independently conducted this coding (GH and SMJ), and any disagreements were resolved via discussion with 2 other independent researchers (SJ and TW) to provide a final quantitative classification for each rating across all apps and domains.

Median scores for professional reviews were calculated for each of the 6 domains. Any scores on or below the median were negative (score 0). Table 1 presents the participant and professional scores.

**Table 1:** Participant and professional scores and their interpretations.

	Participant=0	Participant=1
Professional=0	Negative agreement (both participants and professionals rate negatively)	Professional negative (participants rate positively but professionals rate negatively)
Professional=1	Participant negative (participants rate negatively but professionals rate positively)	Positive agreement (both participants and professionals rate positively)

The primary outcome measure was “participant negative” (participant rated negatively but professional rated positively) across the 6 domains. In addition, we tested participant-professional agreement using the weighted Cohen κ statistic for recommendation ratings and MARS star ratings for all apps for which these ratings were available. Furthermore, 2 PsyberGuide professionals’ recommendations and MARS star ratings were available for each app; therefore, we report the comparison of participant ratings against each professional and an average. We report the Cohen [30] interpretations of the κ values (0.01-0.2, none to slight; 0.21-0.4, fair; 0.41-0.6, moderate; 0.61-0.8, substantial; and 0.81-1, almost perfect agreement). As we asked the participants 2 questions relating to the functionality of the apps (ease of use and difficulties of use), these ratings were both compared with the professionals’ functionality score on PsyberGuide. Professional recommendations and MARS star ratings were not available for Self-Help for Anxiety.

MARS star ratings were compared with app store ratings from the iOS app store and Google Play using Pearson correlations to compare genuine users with lived experiences with app store reviews. All quantitative analyses were performed using SPSS version 27 (IBM Corp) for Windows [31].

#### Qualitative

All open-ended survey responses were thematically analyzed using the Braun and Clarke [32] method, which was also used in previous publications [33,34]. Themes were inductively extracted by 2 researchers (GH and EN) independently, using the analysis framework by Pope et al [35]. This is a 5-stage process and involves (1) familiarizing with raw data, (2) identifying a thematic framework, (3) indexing, (4) charting, and (5) mapping and interpreting—defining concepts, mapping the range and nature of phenomena, and creating typologies. Each of the 2 researchers independently and inductively coded all the participant responses, resulting in 2 thematic frameworks. The 2 researchers then created the final inductive framework together by discussing the similarities and differences between the 2 frameworks and using the elements of the multiple coding approach [36]. Theme names were decided collaboratively by the 2 researchers. Any discrepancies were resolved through discussion between the 2 researchers and were independently checked by a third researcher (SMJ). All qualitative analyses were performed using NVivo version 12 for Windows [37].

## Results

### Sample Characteristics

A total of 21 people participated in the study. Most were women and educated to a degree level, but they were ethnically diverse. Table 2 presents the breakdown of participant characteristics.

**Table 2:** Sample characteristics (N=21).

Characteristic			Participants
**Gender, n (%)**			
	Female	15 (71)	
	Male	5 (24)	
	Nonbinary	1 (5)	
Age (years), mean (SD, range)			29.10 (11.01, 20-60)
**Ethnicity, n (%)**			
	Asian or Asian British	7 (33)	
	Black or Black British	4 (19)	
	White British	6 (29)	
	Other	4 (24)	
**Education status, n (%)**			
	No qualifications	1 (45)	
	A-level	7 (33)	
	Degree	12 (57)	
	PhD	1 (5)	
**Employment status, n (%)**			
	Employed (full or part-time)	10 (48)	
	Student	6 (29)	
	Unemployed	3 (14)	
	Retired	1 (5)	
	Receiving ESA^a^	1 (5)	
Previous use of well-being apps, n (%)			13 (62)
**Average daily digital device use, n (%)**			
	<1 hour	2 (10)	
	1-3 hours	3 (14)	
	3-5 hours	7 (33)	
	>5 hours	9 (43)	
**Most frequently used digital device type, n (%)**			
	Smartphone	13 (62)	
	Desktop	8 (38)	

^a^ESA: employment and support allowance.

### Do Participants Agree With Professionals’ Reviews?

Overall, there was little agreement between the participants’ and professionals’ reviews (Table 3), with most app ratings classified as disagreements (53.2% vs 46.8% agreements). Participants were much less positive about the apps than professionals, with difficulties in use being the most different (Table 4). Multimedia Appendix 2 gives a more detailed account.

**Table 3:** Overall number and relative percentage of negative agreement, positive agreement, professional negative, and participant negative (N=372).

Particulars	Participant=0, n (%)	Participant=1, n (%)
Professional=0	Negative agreement^a^, 76 (20.4)	Professional negative^b^, 149 (40.1)
Professional=1	Participant negative^c^, 49 (13.2)	Positive agreement^d^, 98 (26.3)

^a^Both participants and professionals rate negatively.

^b^Participants rate positively but professionals rate negatively.

^c^Participants rate negatively but professionals rate positively.

^d^Both participants and professionals rate positively.

**Table 4:** The breakdown (number and percentage) of which domains participants scored negatively and professionals scored positively (“participant negatives”; N=49).

Domain	Participant negatives, n (%)
Difficulties of use	17 (27)
Data security	10 (16)
Aesthetics	9 (15)
Ease of use	7 (11)
Perceived impact on well-being	4 (67)
Engagement	2 (3)

### Do Participants Agree With Professionals’ Views?

There was *moderate* to *substantial* agreement between the 2 professionals’ recommendation ratings (Cohen κ_w_=0.667; *P*=.008) and MARS star ratings (Cohen κ_w_=0.571; *P*=.008). However, there was little (*none to slight*) agreement between the participants’ and professionals’ recommendation ratings (Cohen κ_w_=0.048; professional 1, Cohen κ_w_=0.047; professional 2, Cohen κ_w_=0.048). Participants gave lower recommendation ratings on average (mean 3.44, SD 1.09) than the 2 professionals (professional 1: mean 4.22, SD 1.30; professional 2: mean 4.56, SD 1.01). There was also little (*none to slight*) agreement between participants’ and professionals’ MARS star ratings (Cohen κ_w_=0.108; professional 1, Cohen κ_w_=0.124; professional 2, Cohen κ_w_=0.092), with participants again giving lower star ratings on average (mean 3.58, SD 0.91) than the 2 professionals (professional 1: mean 4.44, SD 0.73; professional 2: mean 4.67, SD 0.71).

### Do Participants Agree With App Store Ratings?

Participants’ MARS star ratings of apps were significantly positively correlated with average app store ratings (*r*=0.32; *P*=.01) and with individual iOS app store (*r*=0.27; *P*=.04) and Google Play (*r*=0.31; *P*=.02) ratings. These correlations were low, despite the agreement between iOS app store and Google Play ratings (*r*=0.73; *P*<.001).

### What Do Participants Want From Mental Health Apps?

The thematic analysis of participants’ qualitative data found 11 themes.

#### Cost

One of the most common themes mentioned by respondents was cost. This was largely in response to the survey item “what did you like the least about this app?” All users were able to engage in some content without paying but found it “frustrating to see so many options which you can’t use due to having the free version,” especially when “it wasn’t allowing me to experiment with things and find what’s right for me before purchasing.” Therefore, the most frustrating part of the experience was the hidden costs introduced by freemium or other forms of pricing that provide a limited experience of the app for free, with features behind a paywall, which did not allow participants to try these features without paying. Many participants reported that on the free versions of the apps, there were many adverts, often for users to “upgrade to premium,” which participants found “excessive” and “would ruin the flow or calm I had going.”

#### Aesthetics

The user interface contributed to people’s enjoyment. The structure of app features and layout for each section were the main influencing factors. For example, Insight Timer was described as having a “*professional*” layout, which made the app appear more legitimate. Another was described as clumsy and inconsistent in design, which made it look “like it’s in beta format.” Some colors made the app more engaging, with others providing a “nice and calming” feeling. Some users also felt that their “screen appeared quite crowded due to the number of options,” reducing the appeal.

#### Ease of Use, Navigation, and Functionality

Most apps were described as “very easy and simple” to use and they were able to navigate features “without having to try very hard to find them.” However, prompts to guide navigation to specific features are necessary, especially for users with no prior experience of using well-being apps. Features on the app should also load quickly so users are not irritated. Some apps had technological problems such as glitches, where “one time where it lost my entire journal entry.” This could make the apps difficult for participants to use as “sometimes there [*w*]as content there and at other times, it said ‘We couldn’t find any results.’ I found this quite frustrating.”

#### Interactivity

Participants valued interactivity and particularly enjoyed “daily quotes” and “a voice assistant.” However, interactive prompts, reminders, and notifications also garnered mixed opinions. While some believed the prompts were “useful...for people on their anxiety recovery journey” and “made me actually check in with how I was doing,” others found them unnecessary and “had to turn them off because they got annoying.” These participants wanted the app notification frequency to be optimized so they do not feel they are being nagged.

#### Personalization and Customization

There were mixed responses on the extent to which apps allowed users to have a customized experience. Most participants believed it was an important feature of an enjoyable app experience. Some apps used natural language processing to provide relevant and appropriate responses to user input. Participants, therefore, received “personal insights tailored to you[r] mental health.” The option of customizing color schemes and audio voices was highly favored. Personalization also allowed users to “save your favourite content and create playlists,” “so can reuse particularly helpful courses.”

#### Education and Teaching

Many apps included information in the form of articles and blog posts. Users repeatedly mentioned that this content provided “useful education on mental health issues,” specifically those related to anxiety and depression. Information on managing symptoms and coping mechanisms was especially useful. Participants reported that this educational content helped them understand their own mental health, “[teaching] me more about background of anxiety” and proving participants with “more of a technical understanding of anxiety and stress.” However, it was suggested that this information was not always at a sufficiently deep level of complexity for those with a diagnosis of depression or anxiety (“for someone who already knows about this then it would probably be quite basic”).

#### Tracking and Goal Setting

Apps that provide a clear way to set goals and track progress were said to be interesting and useful. Tracking progress helped participants to “track your mood and identify possible triggers,” and monitoring their mental health “helps me monitor my mind and what triggers me.” Participants found the ability to track anxiety symptoms specifically helpful as it “allows you to understand your anxiety and how it progresses on the graph so you can track progress on the anxiety tracker.” However, other users reported that tracking features caused negative emotions because it was not “helpful or productive to...see the amount of days I potentially felt low.”

#### Variety of Features

Users appreciated a combination of audio and visual content. Apps that require users to perform daily tasks should ensure that those tasks are not repetitive, and some apps should “add more features to attract users.” This often included further developing the existing features (“the check in feature didn’t have enough guidance and was quite bare bones and didn’t provide counter to negative thoughts for instance”). Two participants also requested more features “aimed at the teenage community,” “as there is enough material for adults and kids but I didn’t see as much as I was hoping for teens.” However, a variety of features can be excessive— “sometimes difficult to choose which activity to focus on because there was too much content.” “The huge amount of content stopped it being engaging,” and it was recommended that “they should focus on a few core features.”

#### Data Security and Privacy Concerns

The general sentiment among the respondents was that they had no concerns about the security of their data. This was mainly because they did not enter “anything overly personal.” Others were able to register via their Apple or Google Play account which was perceived as a legitimate process of verification and data protection. Some relied on their prior understanding of UK General Data Protection Regulation regulations to determine the security of their data (“they would have to be complying with the law”). Some reported that although they had seen the declaration on the apps, they thought it should be better signposted (“perhaps a disclaimer can be added to the start of the app making it clear about data security”).

#### Integration With Daily Life

Well-being apps can be used to support users at various points of the day and various locations. Many users were able to schedule times to use them at their own convenience (“specific meditations designed for different times of day—starting the day, commuting, focusing at work”). They also appreciated content relevant to their specific life circumstances (“had a section on dealing with corona which was very useful” and “some exercises to help you cope with specific aspects of life”). Although some participants found “the exercises were nice and short which is very convenient for someone with an insanely busy life like mine,” others found that “as it’s very time consuming, regular use of the app may not [be] sustainable for the long term.”

#### Impact on Well-being

Users reported a change in their well-being, specifically helping people “feel significantly less anxious.” Others appreciated the exercises and courses they engaged with as they were thought-provoking and promoted introspection. Information specifically about mental health status and how to use different strategies to cope with adverse experiences was welcomed. The guided journeys aided them in reducing maladaptive thought processes by helping them understand the origin of their negative thinking patterns which, in turn, helped reduce feelings of anxiety and low mood. Respondents were provided with “exercises to cope with these feelings but also knowledge to understand what anxiety is,” which were greatly appreciated.

## Discussion

### Principal Findings

To the best of our knowledge, this is the first study to investigate how reviews of mental health apps by professionals differ from those by people with mental health problems. Most reviews focus exclusively on professionals’ opinions [38-40], and reviews that appear to be from genuine app users, such as on app stores, are often false [16]. We have demonstrated that these opinions differ, and therefore, professional reviews and those on app stores are not sufficient to provide those with mental health problems the information they want when selecting a mental health app.

We found low levels of agreement among the ratings of professionals, app stores, and people with mental health problems. Participants placed a great deal of importance on app functionality, and most themes generated through the qualitative analysis were related to this aspect. They appreciated a variety of features, which were easy to use, interactive, and with the capacity for personalization [41]. Aesthetics were also very important, as our participants emphasized the importance of a professional layout, with engaging colors and a simple structure. The highest number of participant negatives was for the domain “difficulties of use,” suggesting that current professional ratings are overestimating the ease with which the apps can be used. Overall, we found that more than half (53%) of the app ratings showed disagreements between participants and professionals. This high level of disagreement shows that professionals have highly different views of what is important in a mental health app, compared with those with personal experience of mental health problems.

We found weak positive correlations between app store and participant ratings. This low agreement suggests that ratings of app stores are not representative of the opinions of those who have experienced mental health problems, and therefore, app stores are not sufficient to provide the information desired by those with mental health problems. This discrepancy may be due to the high number of fake reviews in app stores [16], or it may reflect differing priorities between those with lived experiences of mental health problems and laypeople in the general population. We suggest that it would be beneficial for people with lived experiences to rate mental health apps, rather than exclusively professionals, to ensure the ratings are more accurate and representative of mental health service users’ opinions.

### Comparisons With Prior Work

The variety of features our participants preferred mirrors other studies, such as a scoping review of 37 studies on mental health chatbots, which found that usefulness and ease of use were the most frequently assessed features [35]. Importantly, we found that professionals and those with mental health problems disagree in their ratings of mental health apps. This aligns with previous research findings that participants could independently complete less than half of the tasks in apps targeted at chronic conditions [42] and expressed significant frustration with the design features and navigation of the apps. By engaging with those with lived experiences, app designers and professional raters can identify the features of apps that are most important to this population.

Professional raters may also miss some domains that users with lived experiences emphasized. The ability to track and measure their mental health, as well as the provision of informative articles about mental health, was praised by the participants. This replicates other studies. For example, almost three-quarters of people (from a sample where half had experienced mental illness) perceived monitoring or showing progress toward a goal as useful in a mental health app [43]. Our participants’ dislike of excessive notifications was also mirrored in the study by Thornton and Kay-Lambkin [43]. Cost was one of the most frequently mentioned negative aspects of apps, highlighting a major issue with accessibility and inclusivity. Professional reviews on PsyberGuide frequently consider cost in their narrative reviews; however, it is not incorporated in their numerical ratings. Thus, consumers may be influenced by better scores and may fail to note information regarding costs. An alternative approach is that used by the M-Health Index & Navigation Database, which presents each app characteristic or feature as a separate filter [44]. This is beneficial in that it allows consumers to decide which characteristics or features matter to them but is challenging as multiple fields and filters exist. A better understanding of what matters to consumers provides useful information to guide decisions regarding which information to provide and to improve systems providing information to consumers. Apps should be transparent about their costs, rather than hiding features behind a paywall, where it is not possible to evaluate the usefulness of those features before making a payment. This was particularly emphasized in this study, as we had to remove one of the apps from our study as it required users to input credit card details, which would automatically charge an annual subscription, despite offering a free trial. This is a consideration highlighted through the valuable input from the Young Person’s Mental Health Advisory Group [22], reinforcing the importance of patient and public involvement.

### Implications

This study has significant implications for the use of mental health and well-being apps. We show that professionals’ and app store reviews are insufficient for mental health app users to make informed decisions based on the aspects of apps that are important to them. This is even more important in the context of the COVID-19 pandemic, with disrupted usual mental health care and patients relying on web-based mental health support. The study findings suggest that additional work should be conducted to ensure mental health apps are as useful as possible in supporting the public’s mental health. In addition, review platforms should seek to incorporate the views of those with mental health problems when publishing reviews to maximize their relevance for those most likely to use mental health apps.

### Strengths and Limitations

Existing research on what people think about health or mental health apps has focused on the perspectives of predominantly the White population (84%) [45]. Digital tools can help bridge inequalities in access to mental health care; therefore, it is essential to consider the perspectives of typically underserved people. We improved on a prior work with a much more heterogeneous sample (only 28% of our sample was White British). Our sample was skewed toward women (15/21, 71%), but as women are more likely to use mental health apps [46] and the internet for health-related information [47], our sample may be representative of mental health app users in this respect. However, our sample was generally highly educated (13/21, 62%) to degree level. Although smartphone ownership is associated with higher levels of education [48], it is likely that mental health app use and opinions differ based on education, which we were unable to capture in our sample. Future work should aim to investigate differences in reviews of mental health apps with a larger and more diverse sample in terms of gender and educational attainment.

While we investigated whether the participants had prior experience using well-being apps, we did not directly measure whether they had previously used the same apps they used in the study. This may have affected the study; however, as we did not alter the apps in any way, their prior experience may simply corroborate our findings. It is also worth noting that although we refer to these apps as “mental health apps,” the included apps are all general wellness or well-being apps and not digital therapeutics. Distinctions between categories of apps to support the mental health and well-being of consumers are starting to emerge but are still murky as regulations and guidance attempt to catch up with this market. Future work can explore different groups’ understanding of these distinctions to understand what is acceptable for these low-intensity intervention apps.

This study was designed to understand the views of people with experience of mental health problems and so reflects the views of those who are most likely to benefit from the support provided by mental health apps. Our participants had mental health problems, but future work should capture the opinions of a group of people with varying psychiatric diagnoses to understand whether those factors affect app ratings. For example, a randomized controlled trial found that using mental health apps was associated with improvements in depressive symptoms, but they had no effect on anxiety, compared with a control group not using a mental health app [49]. However, most studies found that mental health apps are effective in improving symptoms of both depression and anxiety [1,2,50,51], as well as the quality of sleep [52]. Studies on other conditions, including serious mental illnesses, are limited; therefore, future work should investigate differences in efficacy depending on symptomatology. If differences exist, then there may be differing priorities in mental health apps depending on their diagnoses.

### Conclusions

We found that participants with lived experiences of mental health problems rate apps differently than professionals and that these ratings correlate poorly with those publicly available on app stores. This is particularly important in the current climate of the COVID-19 pandemic, with more people seeking their mental health care on the web. Further research is needed to explore the perspectives of a diverse group of mental health service users. Our participants also emphasized aspects that are not currently captured in the available review systems. Our study findings suggest that aspects such as ease of use, engaging features and designs, low cost, and some educational content should be added in the future.

## Appendix

Multimedia Appendix 1 Predefined protocol for converting qualitative text into the binary classification of participant experience.

Multimedia Appendix 2 Full breakdown of the number of negative agreements, positive agreements, participant negatives, and professional negatives, and their relative percentages, for all domains.

## Abbreviations

MARS: Mobile App Rating Scale

## Data Availability

The data that support the findings of this study can be obtained from the corresponding author, SJ, on reasonable request.
