# Supplementary material for: Comparing Professional and Consumer Ratings of Mental Health Apps: Mixed Methods Study
Source: JMIR Form Res. 2022 Sep 23;6(9):e39813. doi: 10.2196/39813 (PMC9547331; doi:10.2196/39813)
Supplement: Multimedia Appendix 1 [file formative_v6i9e39813_app1.docx]

| **Multimedia Appendix 1.** Pre-defined protocol for converting qualitative text into binary classification of participant experience. | |
| --- | --- |
| **1 = Good** | **0 = Bad** |
| A positive experience | Any negatives mentioned |
| Participants use qualifiers and give more information that **isn’t negative** | Participants use **qualifiers** (moderately, fairly, quite) **and** provide **no more info** |
| Impact on mental health: any positive impact (including with qualifiers e.g. ‘slight improvement’) | Participants use **qualifiers** (moderately, fairly, quite) and **list negatives** |
|  | Suggestions for improvement |
|  | Impact on mental health: no impact or any negative impact |
| Ignore comments relating to different questions | |
