# Supplementary material for: Comparing Professional and Consumer Ratings of Mental Health Apps: Mixed Methods Study
Source: JMIR Form Res. 2022 Sep 23;6(9):e39813. doi: 10.2196/39813 (PMC9547331; doi:10.2196/39813)
Supplement: Multimedia Appendix 2 [file formative_v6i9e39813_app2.docx]

| **Multimedia Appendix 2.** Full breakdown of number of negative agreements, positive agreements, participant negatives, and professional negatives, and their relative percentages, for all domains. | | | |
| --- | --- | --- | --- |
| **Domain and agreement status** | | **N** | **Relative %** |
| **Ease of use** | |  |  |
|  | Negative agreement | 15 | 24.19% |
|  | Positive agreement | 18 | 29.03% |
|  | **Participant negative** | **7** | **11.29%** |
|  | Professional negative | 22 | 35.48% |
| **Difficulties of use** | |  |  |
|  | Negative agreement | 16 | 25.81% |
|  | Positive agreement | 8 | 12.90% |
|  | **Participant negative** | **17** | **27.42%** |
|  | Professional negative | 21 | 33.87% |
| **Aesthetics** | |  |  |
|  | Negative agreement | 11 | 17.74% |
|  | Positive agreement | 12 | 19.35% |
|  | **Participant negative** | **9** | **14.52%** |
|  | Professional negative | 30 | 48.39% |
| **Engagement** | |  |  |
|  | Negative agreement | 19 | 30.65% |
|  | Positive agreement | 3 | 4.84% |
|  | **Participant negative** | **2** | **3.23%** |
|  | Professional negative | 38 | 61.29% |
| **Perceived impact on wellbeing** | |  |  |
|  | Negative agreement | 13 | 20.97% |
|  | Positive agreement | 19 | 30.65% |
|  | **Participant negative** | **4** | **6.45%** |
|  | Professional negative | 26 | 41.94% |
| **Data Security** | |  |  |
|  | Negative agreement | 2 | 3.23% |
|  | Positive agreement | 38 | 61.29% |
|  | **Participant negative** | **10** | **16.13%** |
|  | Professional negative | 12 | 19.35% |
